# Quantum-resilient software security: A fuzzy AHP-based assessment framework in the era of quantum computing

**DOI:** 10.1371/journal.pone.0316274

**Published:** 2024-12-30

**Authors:** Sultan H. Almotiri

**Affiliations:** Cybersecurity Department, College of Computers, Umm Al-Qura University, Makkah City, Kingdom of Saudi Arabia; Shri Ramswaroop Memorial University, INDIA

## Abstract

The introduction of quantum computing has transformed the setting of information technology, bringing both unprecedented opportunities and significant challenges. As quantum technologies continue to evolve, addressing their implications for software security has become an essential area of research. This paradigm change provides an unprecedented chance to strengthen software security from the start, presenting a plethora of novel alternatives. We use a multi-criteria decision-making methodology in this work to evaluate the efficacy of quantum computing approaches in improving software security. As the number of electronic applications grows, software developers strive to produce more sophisticated and user-friendly alternatives. However, in the pursuit of complexity, vulnerabilities may be introduced inadvertently, posing a substantial danger to software security. Our study addresses five major components of the quantum method to overcome these challenges: lattice-based cryptography, fully homomorphic algorithms, quantum key distribution, quantum hash functions, and blind quantum algorithms. The rapid development of quantum bits (qubits) regarded as basic quantum entities adds complexity and risk to the software security landscape. As a result, in the age of quantum computing, evaluating software security becomes not only necessary but also critical. To accomplish this objective, we propose the Fuzzy Analytic Hierarchy Process (F-AHP), a soft computing method, as a reliable tool for accomplishing this goal. Our research aims to prioritise security variables using quantum security criteria, providing an innovative viewpoint on software security evaluation in the quantum computing era.

## 1. Introduction

The existing software systems are now more susceptible and unsecure due to the current computing trends and their growth. In order to solve software security, more effective and special effort must be made in the creation of quantum computers. The most effective strategy for protecting software security from extremely cunning digital attackers is the quantum computing methodology [1]. Significantly, a double-dealing of popular and obscure attacks for which a security risk has existed has led to the bulk of cyber-attacks on networks, software, and web applications. Since software with flaws will likely fail in a very competitive market, software businesses are working harder to secure the security of their product.

**Fig 1:**
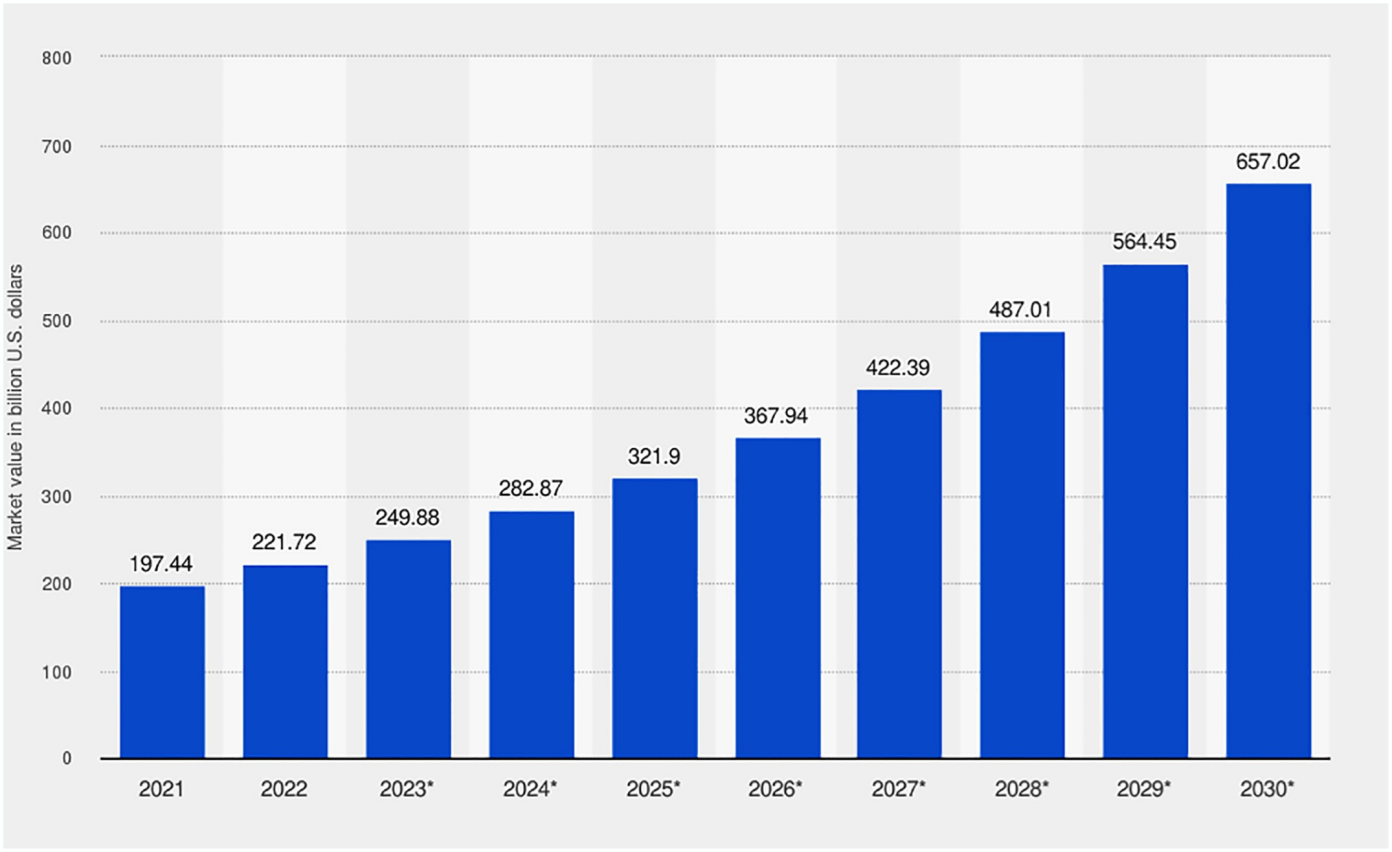
Global cyber-security market size projection: 2021 to 2030 (Measured in billion U.S. dollars) (Source: Next move strategy consulting).

Strong security measures are essential, as the worldwide cyber security market—which was estimated to be worth USD 197.4 billion in 2021—is expected to grow to USD 657.02 billion by 2030. With a compound annual growth rate (CAGR) of 12.8% from 2022 to 2030, this significant growth highlights the changing threat scenario. In the digital age, cyber-security is essential. It includes protecting networks, computers, servers, mobile devices, electronic systems, and data against cybercrimes, cyber-attacks, and cyber-terrorism. Cyber-security refers to a broad approach to stopping a variety of cybercrimes, from identity theft to the planning of highly advanced international digital weaponry. The rising market value emphasizes how urgently strong cyber-security measures are needed to handle the changing threats and weaknesses presented by the internet [2]. The following Fig 1 shows the increasing demand for strong security measures while highlighting the explosive growth of the global cyber security market.

Conceptual design, coding, testing, as well as inquiry are only a few of the stages that make up the life cycle of software enhancement [3]. The phrase “final stage of progress” refers to maintenance. The circumstances in which software is now acceptable can be used to determine software usefulness [4]. For software to be valuable, its advantages should be highlighted. The twenty-first century’s adaptable and changing environment presents fresh difficulties for everyone, including the item [5, 6]. Quantum security in a fully functional quantum computer regulates the amount of security in many quantum alternatives. Modern quantum innovation is advancing at an astounding rate. The Sycamore Processor, created by a team of academics and researchers, is a completely quantum processor that can plan a quantum circuit in 200 s as opposed to tens of thousands of years for a conventional supercomputer [7]. The introduction of the quantum process sycamore had a significant impact on computing. The enormous computational power of sycamore processors enables them to resolve the intricate security mechanisms of software in a variety of security-related contexts.

Software is impacted by the development of security features through quantum security. Both symmetrical and asymmetrical security techniques are used in the traditional computer paradigm. Information is encoded and decoded using a similar key in a symmetric method. In the traditional security mechanism, two or more unique keys are utilised. This is known as an asymmetric approach to security. The security key generated by the Shor algorithm is the only factor used to secure software. The size of the 2042 pieces can be reduced to an unbreakable number [8]. It is possible to calculate out the overall processing time of a typical computer over time. But in essence, a quantum computer can usually separate them in a few minutes or less. The current paper covers the several aspects of software security that could have the biggest impact during the quantum computing era [9, 10]. Our research has examined the ten domains of security as an alternative to the five quantum enabling security approaches. Comparing to other security methods, this field of computing has less research. Future study must take the quantum technique’s design perspective into account.

This study concentrates on current methods, procedures, and security precautions used in software security and quantum computing design; a few examination studies have been done; Scientists have recently concentrated on creating quantum computers, increasing the processing power through different quantum operations [11]. Quantum computing has successfully used sophisticated cryptography calculations to swiftly find solutions. Our work specifically highlights the difficulties and challenges of software security in the quantum age. The key writing sources on quantum computing and product security that we specifically cited while profiling our analysis were described and highlighted [12], referring to the network’s quantum security mechanism and outlining the quicker, more powerful, and more secure interactions between users. Instead of introducing estimations based on mathematical character development, the concept of quantum security is used in cryptography to cover the quantum features of photons under quantum physics. There are number automated signature systems that are susceptible to quantum adversaries, including RSA, El Gamal, hash work, and massive computations for public key encryption. Shore’s observation and quantum computer compromises suggest that the discrepant logarithm problem can answer a sizable chunk of the contemplating problem.

Software security has been evaluated using multi-criteria decision analysis [13]. At the outset, the study prioritises security characteristics over other factors while developing software. The analysis considers five securities in the following level, which has 10 further choices. The product’s useable security was also assessed using the Fuzzy based Analytic Hierarchy Process (AHP) approach [14]. According to a study analysis, a fuzzy-based strategy is more effective and efficient than a traditional one. Additionally, client error assurance is given top attention in the list of chosen qualities. The integrated fuzzy-based approach is the most well-known approach for managing dynamic challenges during software development, claims [15]. Similar to this, security evaluation and plan strategies designated for improving security execution have been highlighted using the fuzzy-based approach to multi-criteria decision-making. In the context for board software development, a fuzzy AHP technique has been provided to evaluate the software’s usability and security [16]. This in-depth analysis suggests that when creating and developing software, developers should focus on the most important software security variables.

The [17] provides information on the most common attacks against meetings of web applications as well as assaults that are aimed at genuine web programme clients when they are constructing a checked meeting that is trusted in web applications. The evaluation gives a study of present security game plans that strategically avoid or lessen distinct hazards by assessing them along four novel files—confirmation, convenience, resemblance, and effortlessness of association. Since people and organisations depend on programming frameworks for their day-to-day operations, it is crucial to deliver secure programming products. In this study, the distribution of secure programming items is investigated, and a paradigm for enhancing programming security is offered. The secure software development life cycle (SSDLC) is a model that integrates software development with security design to guarantee the effective generation of secure programming items. The SSDLC is critical for the production of secure software solutions. In this setting, it is critical to recognize and incorporate SSDLC concepts into software security research and practical application. SSDLC refers to a methodical strategy to incorporating security practices across the software development life cycle, beginning with the original design phases and proceeding through coding, testing, deployment, and maintenance [18]. Developers that follow SSDLC can proactively discover and mitigate security flaws and threats, improving the overall resilience of software solutions. As a result, it is critical to emphasise the incorporation of SSDLC within the research as well as practical components of software security in order to highlight its importance and encourage an integrated strategy to securing software systems in the face of constantly changing dangers such as those posed by quantum computing. Design guidelines are also provided to assist engineers in the development process. In several key areas, we highlight the novelty and contribution of our research work:

- This research study explores the profound impact of quantum computing on soft-ware security, an emerging field with limited prior research, highlighting the need for specialized approaches to safeguard software in this quantum era.
- We explore the tremendous innovations in quantum computing technology, focusing on the game-changing possibilities of quantum processors like the Sycamore Processor, which can execute jobs previously infeasible for classical supercomputers.
- The paper identifies the challenges posed by quantum computing to traditional security techniques, particularly in asymmetric encryption where the Shor algorithm can render existing key sizes ineffective.
- The research study introduces the utilization of multi-criteria decision analysis and the F-AHP to evaluate and prioritize software security features, underlining the superiority of the F-AHP approach for dynamic challenges in software development.

The rest of this study has been organized as follows: Section 3 elaborates on the quantum techniques, and Section 4 enlists the soft computing methodology of fuzzy AHP to determine the weight of the factors. Section 5 discusses computing the weight of the factors in the data analysis section. Section 6 presents the discussion on the findings of this research study. The last section explains the conclusion and future scope in the era of the quantum computer.

## 2. Related works

This section delves into the existing research that serves as the basis for the current investigation on quantum-enhanced software security evaluation. This section looks at a number of researches that explore various facets of quantum computing, how it affects software security, and approaches used to deal with related issues. Together, these studies advance our knowledge of the dynamic environment that the development of quantum technology has shaped. Every study offers insightful information about the rapidly developing subject of quantum computing and its implications for software security, from talks about the possible weaknesses of conventional cryptography systems to investigations of quantum-resistant public-key cyphers. The comprehension of the intricate interactions among quantum computing as well as software security is enhanced by the variety of approaches, which range from systematic mapping studies and cost analyses to fuzzy analytic hierarchy procedures. This review of previous work provides an important foundation for the presentation of this novel approach and contributions to the field of quantum-enhanced software security assessment.

Alyami et al. [19] carried out an extensive investigation in the area of information technology, concentrating on improving software security by means of security algorithms employing quantum computing. The study emphasises how difficult it is to reliably evaluate software security and durability in the face of changing threats. The authors highlight the shortcomings of conventional cryptography techniques in the context of quantum computing and propose a transition to quantum-based security alternatives. To assess and rank security factors, the study uses a symmetrical hybrid approach that combines the Fuzzy Analytic Hierarchy Process (F-AHP) and the Fuzzy Technique for Order of Preference by Similarity to Ideal Solution (FTOPSIS). The study offers important new perspectives on how to manage software durability and security (SSD) when quantum computers are present.

The difficulties brought about by the present technological transformation were discussed by Awan et al. [20], especially as they related to software engineering and the impact of quantum computing technology. Using a fuzzy AHP, the study identifies, investigates, and ranks the most important issues facing the software sector. After conducting a thorough analysis of the literature, the authors determined that institutional and organisational impediments constituted the biggest obstacles to the widespread adoption of quantum computing. The results highlight the nascent stage of quantum computing research and highlight the necessity of utilising multidisciplinary methodologies to tackle recognised obstacles.

Alyami et al. [21] examined the definition and nature of quantum computing in relation to software security, emphasising how existing cryptosystems would soon become obsolete in the face of powerful quantum computers. The paper focuses on the level of quality of "Software durability" as a crucial factor impacted by quantum computing as well as highlights the need for extensive research in quantum cyber security. The researchers suggest a comprehensive analysis of security aspects that will have a major influence on software longevity in the quantum era.

A cost investigation regarding applying Grover’s key search attack on the KATAN block cypher was provided by Rahman & Paul [22] in relation to NIST’s standardisation of post-quantum cryptography. The goal of the research is to create minimally-deep reversible quantum circuits using and gates and Toffoli gates. The study adds knowledge about how to implement quantum algorithms with little resources while adhering to NIST’s depth constraints.

A thorough mapping research of quantum software testing engineering was carried out by García de la Barrera et al. [23], offering a comprehensive picture of the state of the art at the moment. The research emphasised the value of reversible circuit testing and found trends in testing methods, such as statistical methods and Hoare-like logics. The results aid in comprehending the environment surrounding quantum software testing procedures.

The goal of Almotiri et al.’s study [24] was to evaluate healthcare software’s (HS) security during quantum computing activities. In order to assess quantum security algorithms, the study suggested a hybrid approach that combines the Fuzzy Analytic Hierarchy Process (F-AHP) with the Order of Preference by Similarity to an Ideal Solution (TOPSIS) method. The study highlights the reliability and practicality of the hybrid technique for assessing healthcare software security as well as offers suggestions for building a safe HS.

In order to handle the dangers related to less reliable quantum computers as well as workload scheduling methods, Phalak et al. [25] proposed Quantum Physically Unclonable Functions (QuPUFs). The study’s experiments on actual quantum hardware show how well the suggested QuPUFs work to achieve high inter-die Hamming Distances.

Raavi and colleagues [26] investigated the threats that quantum computing presents to public-key cryptography security. The study compared the effectiveness and security of Dilithium, Falcon, and Rainbow—three of the NIST finalist post-quantum cyphers. In order to quantify security strengths, the research established the depth-width cost for quantum circuits (DW cost). It also carried out a thorough analysis to guide the design and standardisation of post-quantum cyphers.

In response to growing concerns about the potential impact of quantum computing on communication security, Pedone et al. [27] proposed a comprehensive software stack designed to facilitate the integration of Quantum Key Distribution (QKD) into modern distributed infrastructures. Their research introduced a QKD simulator as a pivotal tool for evaluating QKD protocols and identifying practical enhancements to these mechanisms. By simulating real-world scenarios, the study provided valuable insights into adapting quantum cryptographic methods to align seamlessly with contemporary virtualization frameworks and cloud-based paradigms. This approach underscores the importance of bridging quantum cryptography with existing technological ecosystems, ensuring that emerging quantum solutions can effectively address the security challenges posed by quantum advancements. Table 1 presents a meta-analysis of recent related works, highlighting key advancements and their relevance to this study.

**Table 1:** Meta-analysis of some recent related works.

Reference	Focus	Methodology	Key Findings
Alyami et al. [19]	Enhancing software security with quantum computing	Symmetrical hybrid technique: F-AHP and FTOPSIS	Quantum-based security alternatives needed
			F-AHP and FTOPSIS effective for SSD
Awan et al. [20]	Challenges in software industry due to quantum computing	F-AHP for identifying and prioritizing challenges	Institutional and organizational barriers identified
			Interdisciplinary research needed
Alyami et al. [21]	Quantum computing’s impact on software security	Thorough assessment of software durability	Cryptosystems may collapse with large quantum computers
			Emphasis on intensive quantum cyber security research
Rahman & Paul [22]	Cost analysis of Grover’s key search attack on KATAN	Reversible quantum circuits, cost analysis with ProjectQ	Designs using and gates and Toffoli gates more resource efficient Respects NIST’s depth restrictions
García de la Barrera et al. [23]	Systematic mapping study of quantum software testing	Identification of trends in testing techniques	Statistical approaches and Hoare-like logics prevalent
			Reversible circuit testing relevant
Almotiri et al. [24]	Assessment of healthcare software security during quantum computing	Hybrid solution: F-AHP and TOPSIS for quantum security algorithms	Recommendations for creating secure healthcare software
			Accuracy of hybrid technique for security assessment
Phalak et al. [25]	Threats in quantum cloud computing, QuPUFs	Proposal and experiments on QuPUFs	QuPUFs mitigate risks from less-trustworthy quantum computers Experiments on real quantum hardware demonstrate effectiveness
Raavi et al. [26]	Security and performance comparison of post-quantum ciphers	Depth-width cost for quantum circuits, analysis in Quantum Annealing	Dilithium, Falcon, and Rainbow compared for security and performance
			DW cost introduced for measuring security strengths
Pedone et al. [27]	Integrating Quantum Key Distribution in modern infrastructures	Software stack for QKD integration, QKD simulator	Adapting QKD to cloud and virtualization paradigms
			QKD simulator for testing protocols and identifying enhancements
This Research	Quantum-enhanced software security assessment	Fuzzy Analytic Hierarchy Process (F-AHP)	Prioritizing security variables in quantum computing era
			Contribution to future SSD research

A clear research gap in the corpus of work related to quantum-enhanced software security assessment has been identified by the literature survey. Even while earlier research has looked into the difficulties that quantum computing poses for software security and has put forth a number of different approaches, there is still a clear need for a unified, all-encompassing strategy that puts security factors first in the context of quantum computing. The research gap that has been highlighted demonstrates the lack of a unified approach to assess software security in light of quantum developments. In order to bridge this gap, this work suggests a novel use of the Fuzzy Analytic Hierarchy Process (F-AHP) as a trustworthy method for ranking security factors affected by quantum computing. By adding F-AHP to the evaluation framework, we hope to provide a methodical and efficient way to analyse software security in the quantum era and add an insightful viewpoint to the ongoing discussion on quantum-enhanced software security.

In our research study, we make use of computing’s revolutionary effects on information technology, especially as it relates to quantum computing. The advent of unbreakable program design in the age of the quantum paradigm has opened up a wider range of improvised alternatives for increasing software security. As the need for complex yet user-friendly electronic systems grows, developers must contend with the difficulty of upholding software security amidst growing complexity. In order to thoroughly assess the quantum computing technology, our study uses a multi-criteria decision-making selection process. Lattice-based encryption, completely homomorphic algorithms, quantum key distribution, quantum hash functions, and the blind quantum algorithm are the fundamental elements of this quantum approach. Qubits, the core components of quantum computing, are proliferating quickly, but this poses concerns to the security environment. As a result, evaluating software security is crucial in the age of the quantum computer. A key tactic in this situation is the skillful implementation of the fuzzy analytic hierarchy process (F-AHP) soft computing method. Our current research diligently works towards a parallel goal, utilizing quantum security standards to accurately assess the importance of various security factors.

## 3. Quantum techniques

The efficiency of current cryptographic computations is sufficient, but the development of quantum time would call for more reliable techniques. In the quantum revamped scenario, the key will be the ability to obtain the cryptography cycle data. Quantum key allocation, a new even arrangement of association encryption, employs the problematic weak rule to deny developers access to the data [23]. The use of techniques like the lattice-based cryptographic algorithm (LBCA), the fully homomorphic algorithm (FHA), quantum key distribution (QKD), the quantum hash function (QHF), and the blind quantum algorithm (BQA) is essential for the security of software and web applications. These procedures control various quantum allocation keys. The security of the software is ensured by the usage of protocols. Quantum cryptography will ensure software security. The operational efficiency of the current cryptography method is weak due to the development of quantum computing. This creates a considerable challenge. A few quantum-enabled cryptographic techniques can be utilized to get around this problem.

### 3.1. Lattice-based cryptographic algorithm [C1]

**Fig 2:**
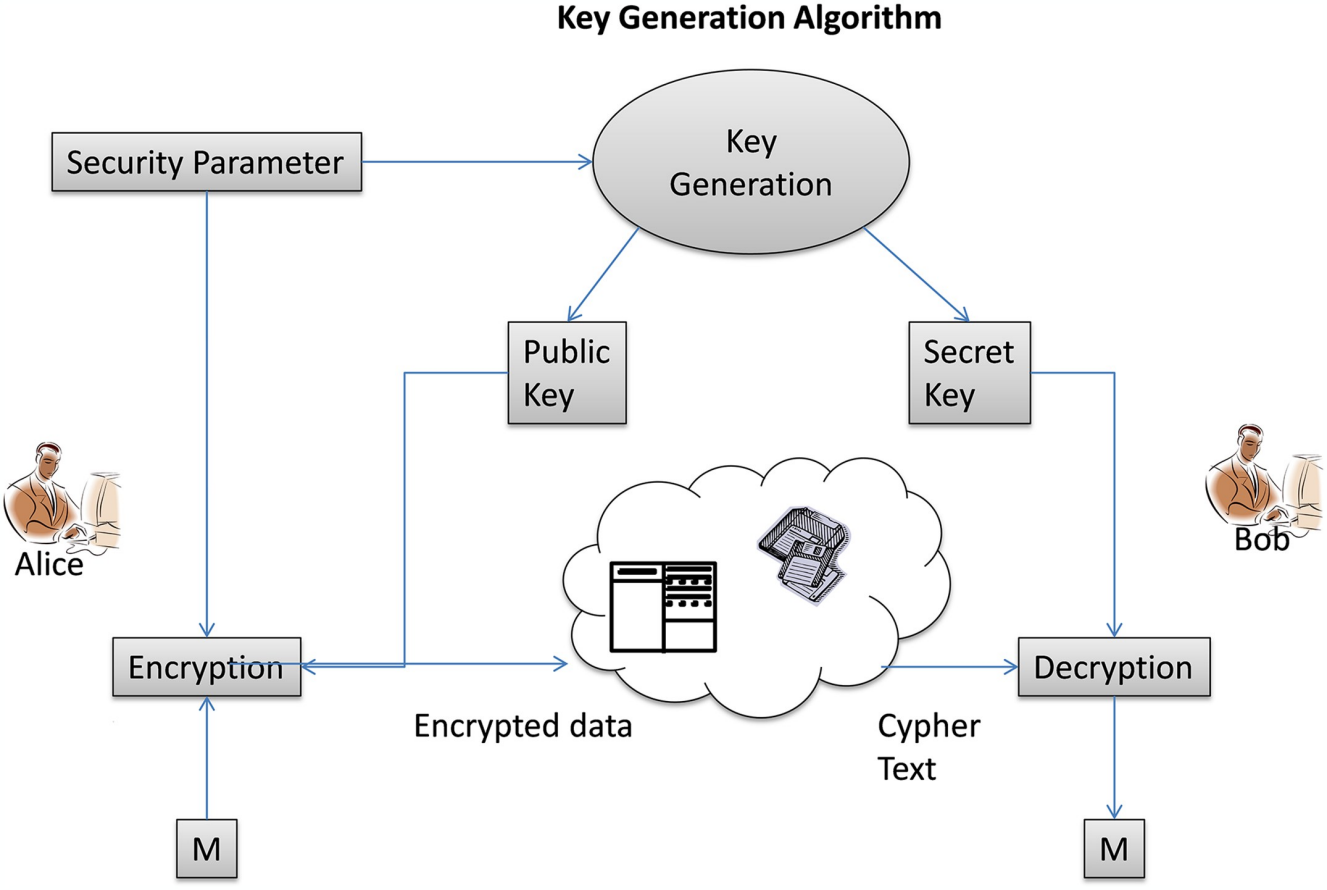
Block diagram of lattice-based cryptography.

The security of information against quantum computing is ensured by the post-quantum cryptography technique in issue. Since its creation by [24], lattice-based encryption has remained impenetrable; the n-dimensional vector produces a collection of vectors in the n-dimensional periodic space that makes up the lattice structure. The term “basic lattices” [25] is often used to describe them. The multidimensional lattice structure is shown in Fig 2. The difficulties that lattice-based cryptography encounters are the shortest vector problem (SVP) and the LLL algorithm. SVP uses a random input lattice that has some approximation. The LLL algorithm was first presented in 1982 by the researcher Lenstra. The approximate speed of this technique is in2(O(n)), where n is the lattice’s size.

Lattice-based cryptography requires the use of four different methods, namely setup, key generation, encryption, and decryption. Fig 2 shows the many stages of the various given jobs in lattice-based cryptography. First, the setup calculation accepts the security boundaries as the input and, as a result, returns the public bounds. The computation of the key generation is finished after accounting for the effect of the stage or setup. It requests data on public boundaries and then outputs the public and cypher keys as a result. The information is encrypted, and the plaintext is scrambled using the donation for the public key Alice. The cypher text is finally cracked within a public cloud by taking the secret key out of the hazardous region and looking at the plaintext-decoded data. The concealed hyper-plane problem’s one-way secret entrance potential can be exploited to create a cryptosystem whose security verification is only reliant on the worst-case scenario. The massive cypher text development during this process led to the consideration of worst-case scenario case drops as well as the key sizes required for this cryptosystem to provide a high enough level of security designed to be a feasible replacement for existing cryptosystems at all times. Additionally, this cryptosystem was the target of a heuristic threat. Since then, this fundamental guidance has been enhanced and prompted.

### 3.2. Fully homomorphic algorithm [C2]

**Fig 3:**
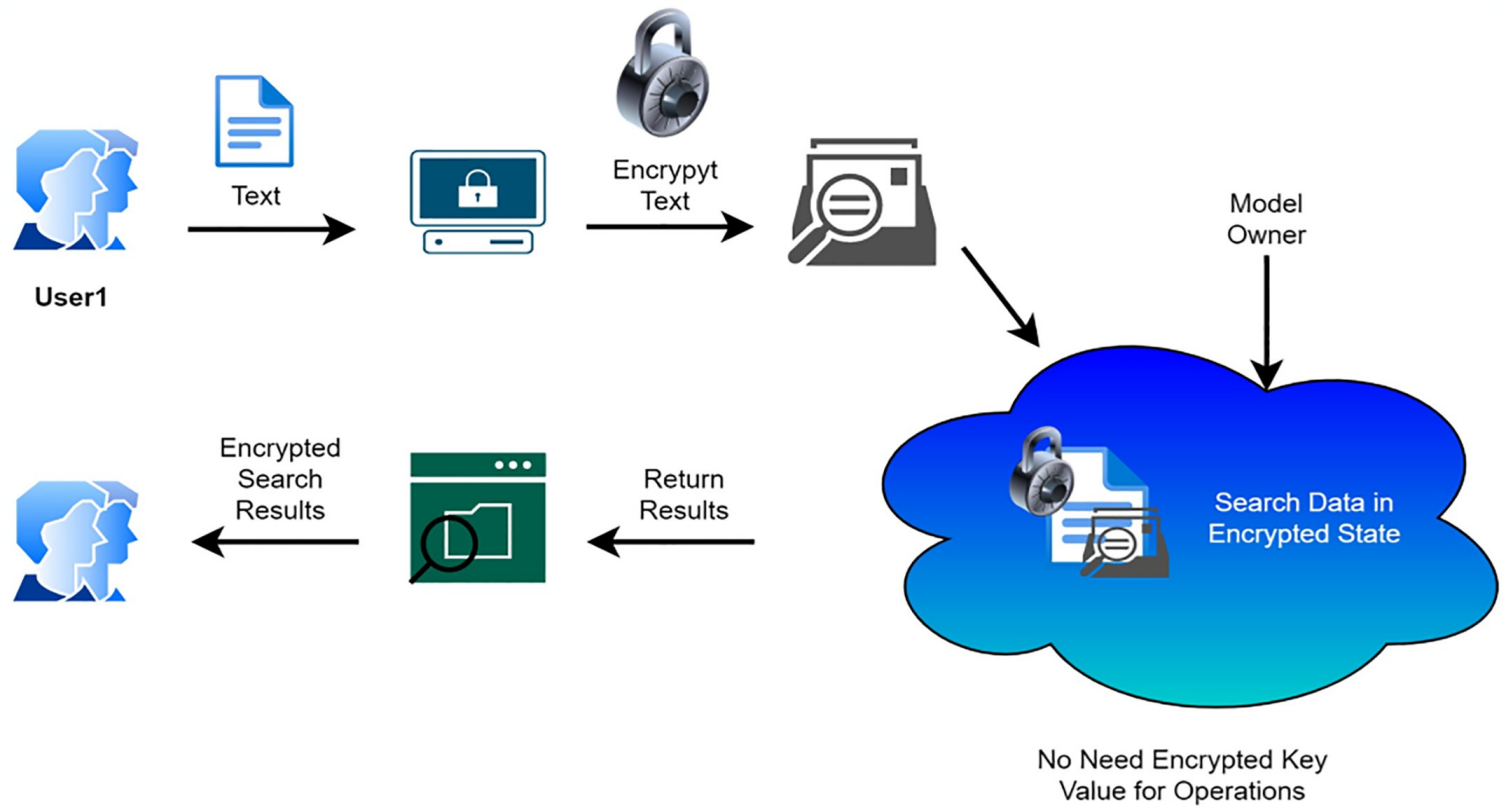
Block description of homomorphic encryption.

Anyone can control information exchange between two organizations without disclosing it thanks to fully homomorphic encryption [26]. We can better understand this concept by using the example of a political decision-making process. The two main parties or partners in a political campaign are the voters and the candidates that the electorate chooses to support. The political decision-making commission, an outsider in this situation, is able to count the anticipated number of votes for each pioneer but is unable to do it before the scheduled time, Therefore, even if the material is publicly accessible, homomorphic encryption prohibits outsiders from decrypting it [27]. Experts in post-quantum cryptography have not yet found a solution for the homomorphic encryption security mechanism that quantum technology enables. The homomorphic encryption technique benefits from key exchange. The homomorphic encryption algorithm scrambles the information using the public key, and mathematical skills are needed to decipher and unscramble the calculation. The function decodes the data’s security, and it cannot be decrypted at all. Fig 3 displays the homomorphic encryption block diagram.

### 3.3. Quantum key distribution [C3]

**Fig 4:**
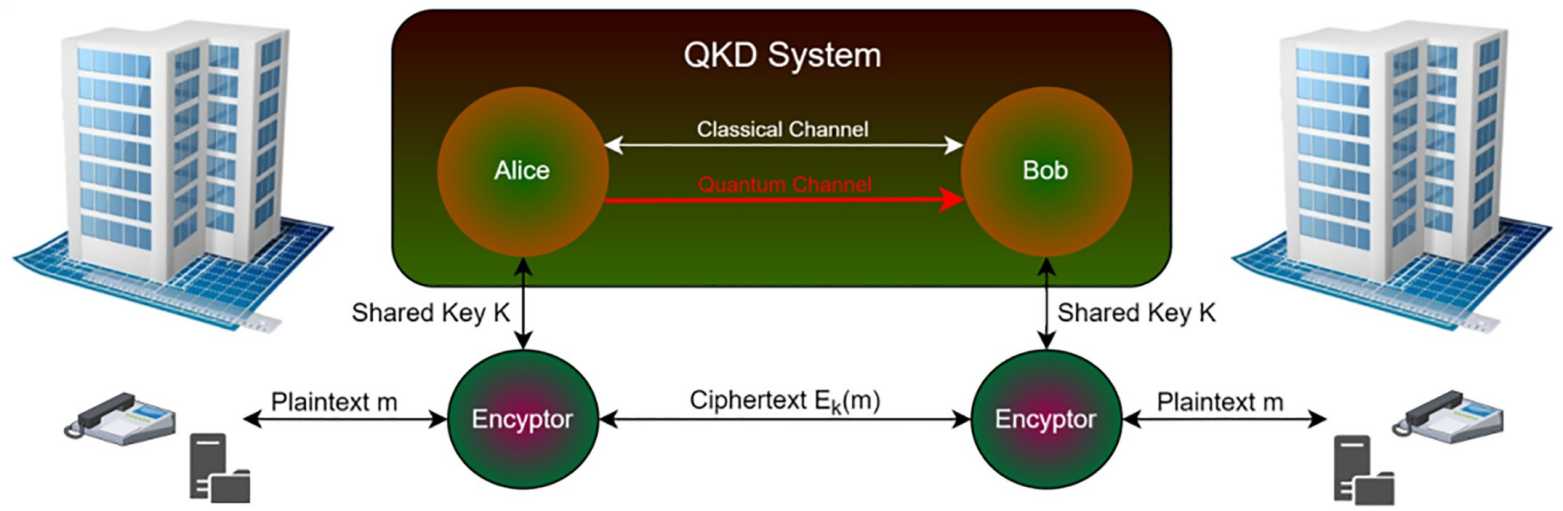
Schematic diagram of quantum key distribution algorithm.

In the framework of quantum key distribution, QKD, which behaves differently from traditional computer architecture, it is utilized to convey encryption key information [28]. It is then feasible to move the data transfer in the quantum key conveyance from the existing fiber-optic line, which was previously restricted to a different fiber-optic line. As a result, correspondence is less expensive. In light of satellite correspondence, there is one additional correspondence. The foundation of this correspondence technique, commonly referred to as the “scary action a long way away”, is an Einstein premise [29]. In recent years, China has started experimenting with quantum correspondence satellites. The corresponding rule is the trapping, or cycle, in which the photons turn alone. The point at which both have a connection is the turning of the photons and our correlation with it. The ability to generate erratic numbers that can be used in encryption calculations, if they are not conversing with one another over this connection, is what this means. This encryption procedure is very expensive, as shown in Fig 4.

### 3.4. Quantum hash function [C4]

A quantum hash capability is any computational restriction that converts an emotional line of data into a yield of a fixed length. Whether it is names, social security numbers for retirement, mp3 files, or anything else, we sincerely want to condense all information into fixed-length words. To solve this, we require deterministic, open, pseudorandom hashing capabilities. We must realize that the hash of any given piece of data, “x,” will always be identical; in other words, the hash is computationally constrained by how it is executed. To enable anyone to use it, we want the execution to be made public.

**Fig 5:**
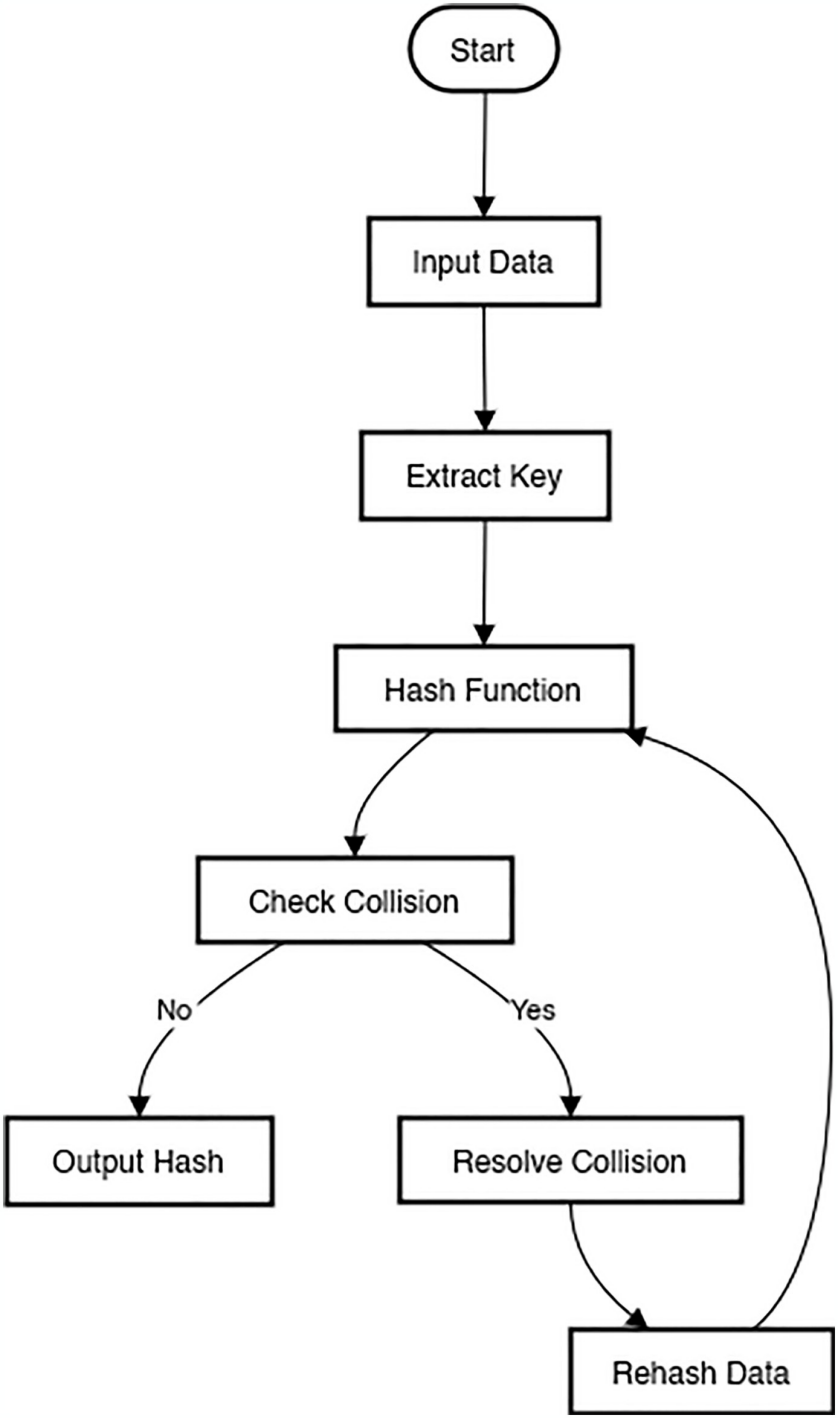
Flow chart diagram of quantum hash function.

In a similar vein, we should disguise the primary commitment by making the yield appear irregular. A hash capability can be implemented using a few crucial properties. Hash works are quite valuable because they are what makes them up. Quantum cryptography uses the fundamental requirement for a single-direction hash capability. Think about the hash function, whose output is 128 bits long. It should be easy to register the hash for x, but it should be impossible or impossibly difficult to reverse the hash and locate x. The 2^128^ possible yields, or more than 340 trillion possibilities, are predicted [9]. Almost anything can be handled by a 128-digit hash [30]. Fig 5 shows the flow chart diagram of a Quantum Hash Function, specifying the sequential steps involved in its operation. This diagram provides a visual representation of the process for implementing and analyzing quantum hash functions in cryptographic applications.

### 3.5. Blind quantum algorithm [C5]

The blind quantum (BQ) approach allows a client to execute a quantum computation while concealing the operation’s design details. Although BQ standards aim to ensure only computation security, many people consider checking the computation as it is being performed by incorporating hidden tests within the calculation. The BQ algorithm is a quantum computing and quantum encryption algorithm [31]. Assume a client does not own a sophisticated quantum device and wishes to hire a server with a flexible, forgiving quantum computer. The BQ algorithm safely routes a client’s information, yield, and algorithm data to the server’s quantum computer. The crucial consideration of the estimation-based quantum computation will be used in the BQ algorithm [32]. It defines a group state as generated as a snare’s asset and afterward. There is a series of single-qubit estimations. One needs to adjust points depending on the calculation. A single qubit estimates the nature of the single qubit. The bunch state is independent of the algorithmic judgment. It is unlikely that the server would send the client a lot of states. Additionally, the client executes the single-qubit estimates, and the server does not obtain any data from either the nuances or the outcome of the client’s computation. The no-flagging rule guarantees the protocol’s security.

**Fig 6:**
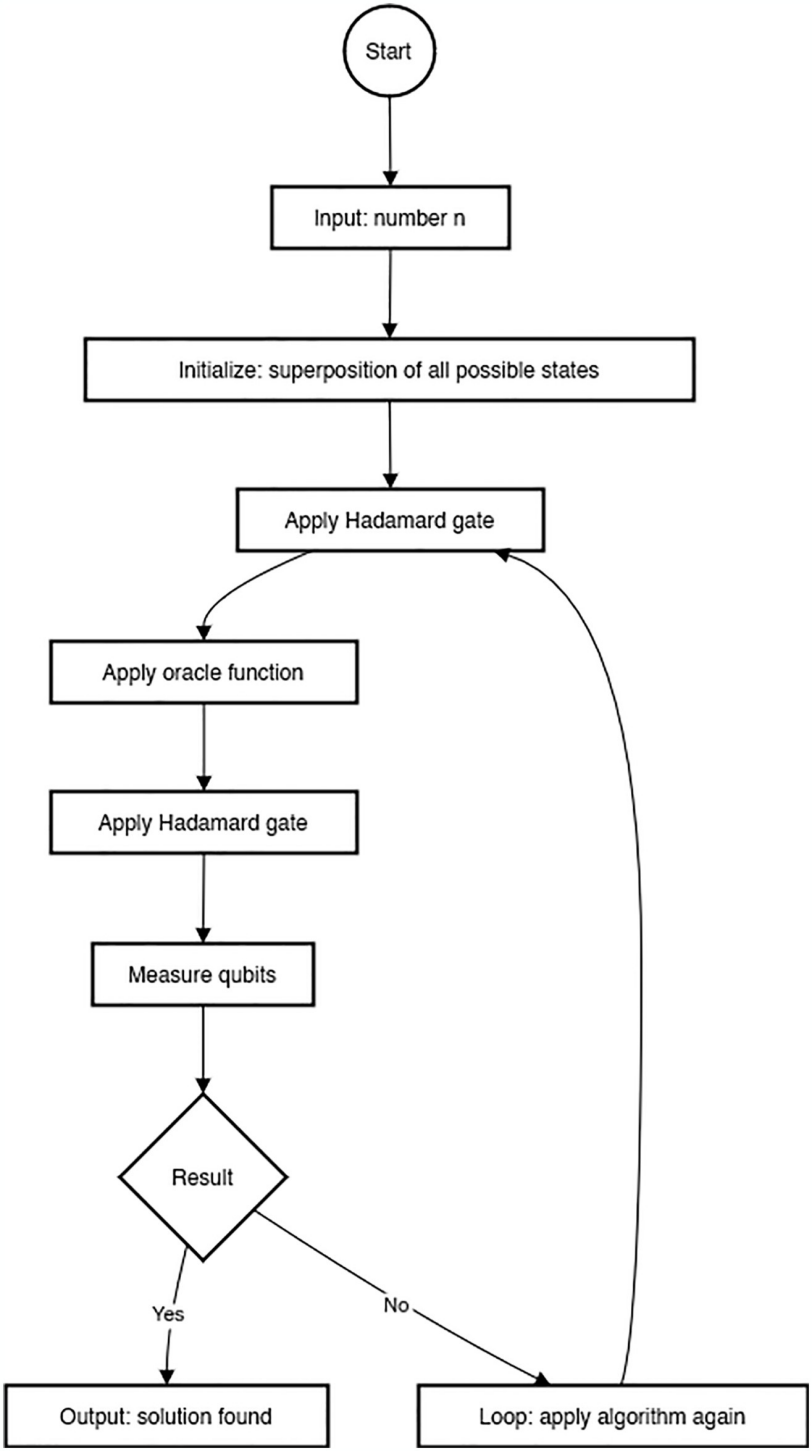
Flow chart diagram of blind quantum algorithm.

The BQ method may be implemented by using a good unitary activity to provide information that encodes the complete circuit to be evaluated as well as any pertinent information states. This establishes a strong connection to the calculation using encoded data, a system similar to calculations for visually challenged people where the evaluated activity is public. The BQ approaches in software security are a new dimension used to secure software programs. The blind quantum algorithm, which enables Alice, who lacks sufficient quantum innovation, to assign her quantum security to Sway, who has a fully functional quantum computer, and it prevents Bounce from learning about Alice’s feedback, result, and calculation. This is another safe quantum computing security technique. Traditionally, Alice needs a machine that can generate quantum states, like single-photon states. This study suggests a new computing convention where Alice only makes estimates, like polarization estimations with a limit identifier. Estimating a state is much easier to comprehend than the age of a single qubit state in some exploratory arrangements, such as optical frameworks. In this way, our customs make Alice’s weight easier. Additionally, the no-flagging standard, which is more important than quantum physics, is what ensures the security of our convention. Our conventions are finally gadget-free; therefore, Alice no longer needs to rely on her estimating device to ensure security [33]. Fig 6 depicts the flow chart diagram of the Blind Quantum Algorithm, outlining the steps for quantum computation with privacy-preserving techniques. The diagram highlights the process of executing quantum operations while maintaining the confidentiality of the data.

Lattice-based cryptography, famed for its impenetrability, provides a strong defense against quantum computing threats. However, its computational requirements can be substantial, especially when coping with higher lattice sizes. The key benefit is its resistance to quantum assaults, which makes it a viable post-quantum cryptography solution. Fully homomorphic encryption provides a one-of-a-kind capacity to execute actions on encrypted data without decryption. This attribute protects data privacy but adds computational overhead. In the setting of quantum computing, its security remains unrivalled, making it a crucial tool for safe data processing. Quantum key distribution makes use of quantum features to provide a secure key exchange while also providing cost-effective and tamper-evident communication links. However, implementation can be costly, particularly in satellite-based settings. Quantum key distribution relies on the fundamental principles of quantum physics, ensuring strong security against eavesdropping.

Data integrity as well as authentication rely heavily on quantum hash algorithms. They offer deterministic, public, and pseudorandom hashing. The key problem is to keep the output looking random while remaining computationally efficient. These hash functions are critical in quantum cryptography, but they must be carefully designed to effectively prevent quantum assaults. Blind quantum algorithms allow for secure quantum computations while obscuring the operation’s details. While assuring computing security, they may introduce additional complexity, such as the requirement for the production of quantum states. The protocol’s security is ensured by the “no-flagging” principle, making it a vital addition to quantum computing security.

Therefore, these quantum security methods provide significant advantages in the face of quantum attacks. Post-quantum security is provided by lattice-based cryptography. Fully homomorphic encryption ensures privacy-preserving computing, tamper-evident communication with quantum keys, data integrity with quantum hash functions, and safe quantum computations with blind quantum algorithms. However, each approach has computational challenges as well as potential complexity that must be carefully considered when applied to specific security circumstances.

## 4. Methodology

**Fig 7:**
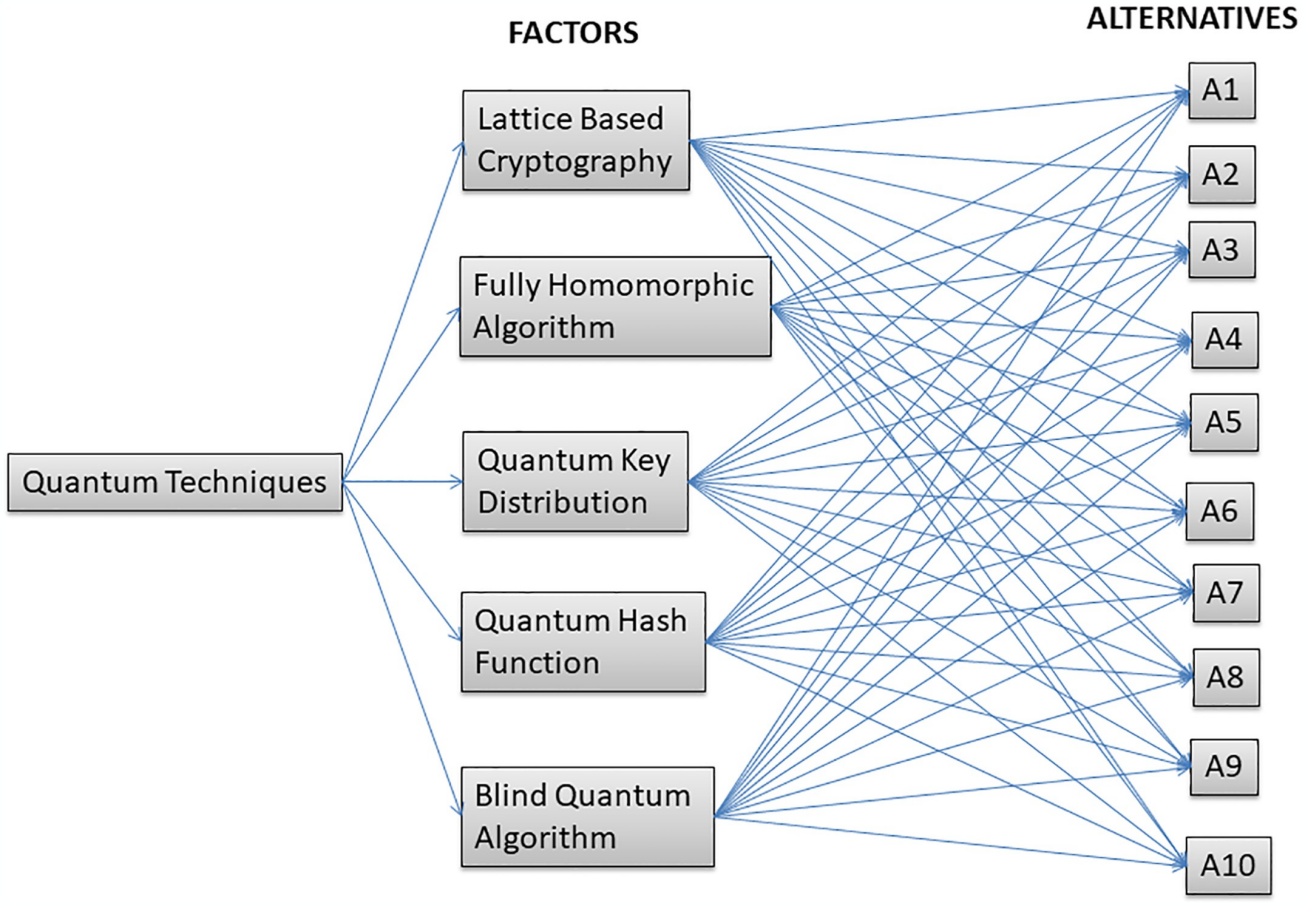
Hierarchy of quantum techniques and alternatives.

This section explains the F-AHP approach of multi-criteria decision-making. This is a classical and fuzzified methodology for identifying the impact of factors during the quantum-enabled computing that could break the network’s security mechanism and the software [8]. From alternatives A1 to A10, we created a hierarchy diagram that depicts the relationship between quantum technique factors and alternatives of software security. The Fig 7 shows the hierarchy of quantum techniques and alternatives, showcasing the relationship and comparative positioning of various quantum methods within the broader context of quantum technology applications.

### 4.1. Fuzzy Analytic Hierarchy Process (F-AHP)

F-AHP introduces a fuzzy hypothesis of the basic scientific order cycle developed by [34]. AHP is a dynamic scheme usually adopted in diverse multi-standard dynamic problems. F-AHP is particularly applicable in this research study because it has the capability to handle intrinsic uncertainties and complexities inherent in decision-making upon evaluation of quantum-resilient software security. Unlike other MCDM approaches, F-AHP embeds fuzzy logic into its model to capture imprecision and subjective judgments that are quite evident in security assessments within emerging fields like quantum computing. This will only make the F-AHP more adaptable to the nuanced and developing natures of quantum technologies for a more refined and accurate prioritization of security factors. The structure of F-AHP allows the systematic decomposition of complex problems into manageable components, allowing for clarity with respect to how various quantum techniques contribute toward overall security. Contrasting that, a large number of MCDM methods cannot provide this flexibility or have to struggle with the quantum criteria of such an abstract nature. That is why F-AHP stands out when trying to capture and analyze the multifaceted aspects of quantum-resilient security solutions.

It works through pair-wise comparisons of different choices having distinct and different rules, furnishing a choice-assistance device for multimodel choice problems. Selection of criteria is the first step of the F-AHP process, which involves the identification of relevant security factors; in this instance, prioritization is done by importance regarding quantum resilient software security. This further involves pairwise comparisons where each criterion will be compared with others to establish relative significance. A comparison thus yields a matrix used in calculating weights for each criterion. This latter step is involved with the integration of fuzzy logic controls for having intrinsic uncertainty and subjective judgment in the process of evaluation for allowing discretionary decisions. This integration of fuzzy logic involves the application of membership functions and fuzzy operators to aggregate the data of comparison and its analysis. Each of these steps is elaborated on in this section for better understanding of the F-AHP methodology in evaluating quantum-enhanced security measures.

In an overall AHP model, the primary level is the aim, and the second and third levels are those levels where the standards and sub-measures are located individually. Finally, the possibilities become perceived at level 4 [35]. Essential AHP has benefited from the fuzzy rationale approach since it does not preclude ambiguity for individual decisions. In F-AHP, the semantic factors, which are handled by three-sided numbers, are used to undertake pairwise evaluations of the two rules and the alternatives [24]. One of the initial uses of fuzzy AHP was carried out by [36]. They described the pair-wise correlations’ three-sided involvement capabilities. Later, deciding the requirements of the examination proportions with three-sided participation capabilities [37], contributed to the subject. In addition, ref. [38] proposed another method for using three-sided numbers in pair-wise comparisons. Although a few extra tactics are used in F-AHP, this review aims to determine the overall significant loads for the models and the other alternatives [39, 40].

**Fig 8:**
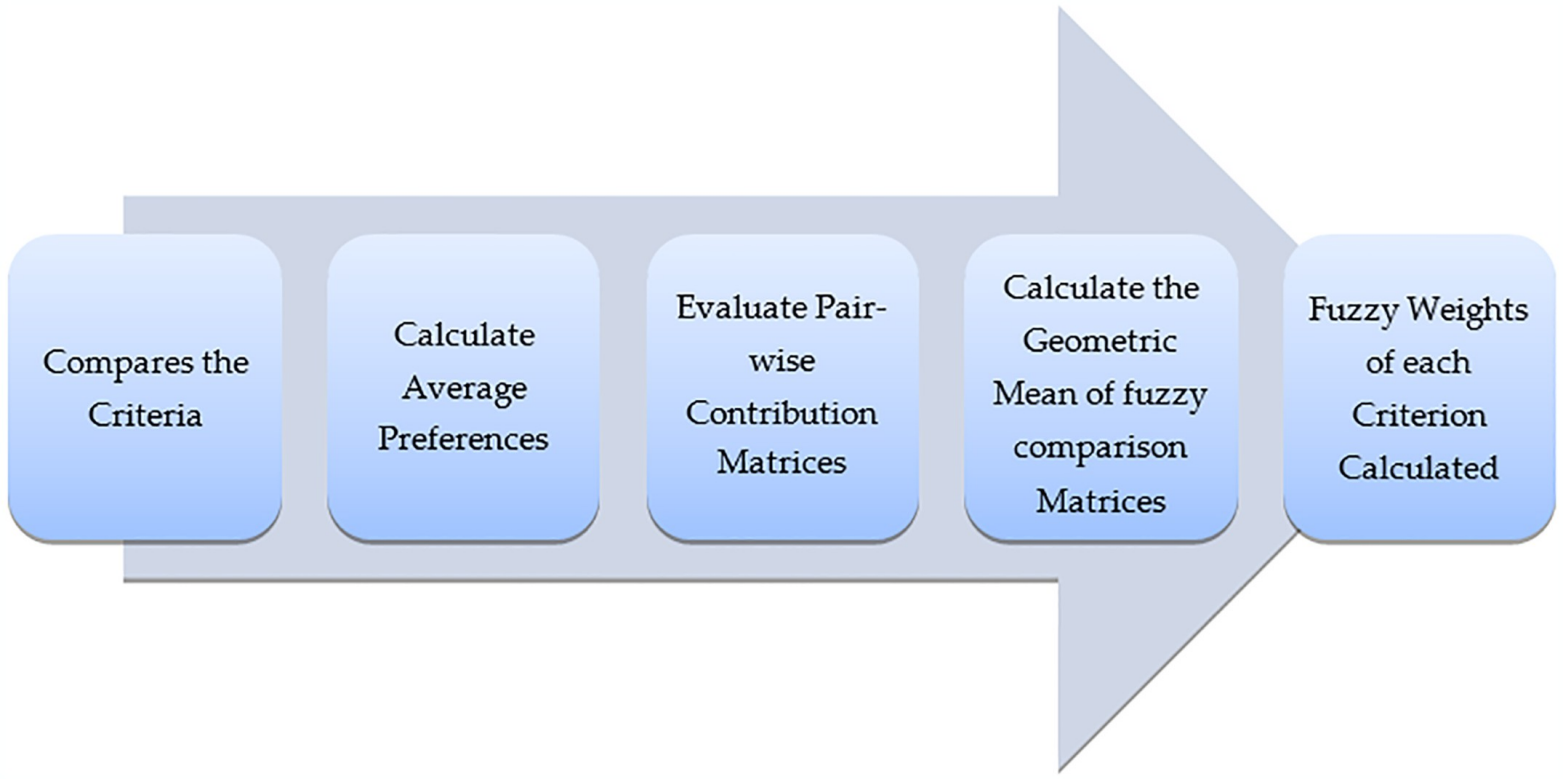
Fuzzy AHP process.

Addressing empirical validation needs for the Fuzzy AHP methodology involves drawing from insights of a holistic literature review and analysis of existing studies. In this regard, the study considers those that have investigated the issue at the intersection between quantum computing and software security, focusing on empirical evidence and practical applications. Alyami et al. [19] followed up with another relevant contribution to quantum-enhanced software security, putting forward a hybrid approach of Fuzzy AHP with FTOPSIS and presenting the practical applicability of the methodology. Empirical studies by Awan et al. [20] and Almotiri et al. [24] also established the effectiveness of Fuzzy AHP in the identification and analysis of crucial challenges and security measures, respectively. These works show the amenability and validity of the proposed methodology in actual scenarios. Consolidating these empirical results, this study stands on a strong base of precedent work with respect to the effectiveness of the Fuzzy AHP framework in the evaluation of quantum-resistant software security. The steps of the multi-criteria decision analysis of the fuzzy AHP procedure are listed below in Fig 8.

The real-time problems are based on criteria. Accurate decision-making can be a major problem in diverse and complex choices. The base of artificial intelligence is the decision of the criteria. The different approaches are used to convert the linguistic values into triangular fuzzy numbers (TFNs). The values are between 0 and 1 and use the Saaty scale. The computational ease of the TFN membership function and its use with fuzzy data are the driving forces behind this adoption [41, 42].

In the fuzzy AHP model, we aim to incorporate uncertainty and imprecision in decision-making. This is achieved by assigning fuzzy membership functions to the elements of the decision hierarchy. In the context of the fuzzy AHP model, Eq (1) assigns a membership value, denoted as β_b (s), within the range [0,1].


$$\beta \_\text{b}\;\left(\text{s}\right)=\text{a}\to \left[0,1\right]$$ (1)

Eq (2) defines β_b (s) using piecewise functions as follows:

$$\beta \_\text{b}\;\left(\text{s}\right)=\{\text{s}/\left(\text{bi}-\text{c}\right)-\text{c}/\left(\text{bi}-\text{c}\right)\;\text{s}\in \left[\text{c},\text{bi}\right]\text{s}/\left(\text{bi}-\text{d}\right)-\text{d}/\left(\text{bi}-\text{d}\right)\;\text{s}\in \left[\text{bi},\text{d}\right]$$ (2)

In Eq (2), the variable “s” represents the membership degree, indicating the extent to which an element belongs to a particular linguistic term within the triangular membership function. The variable “c” represents the lower bound of the triangular membership function, defining the point where the membership value starts to increase from 0 to 1 as “s” moves from “c” towards “bi”, which is the middle bound.

**Fig 9:**
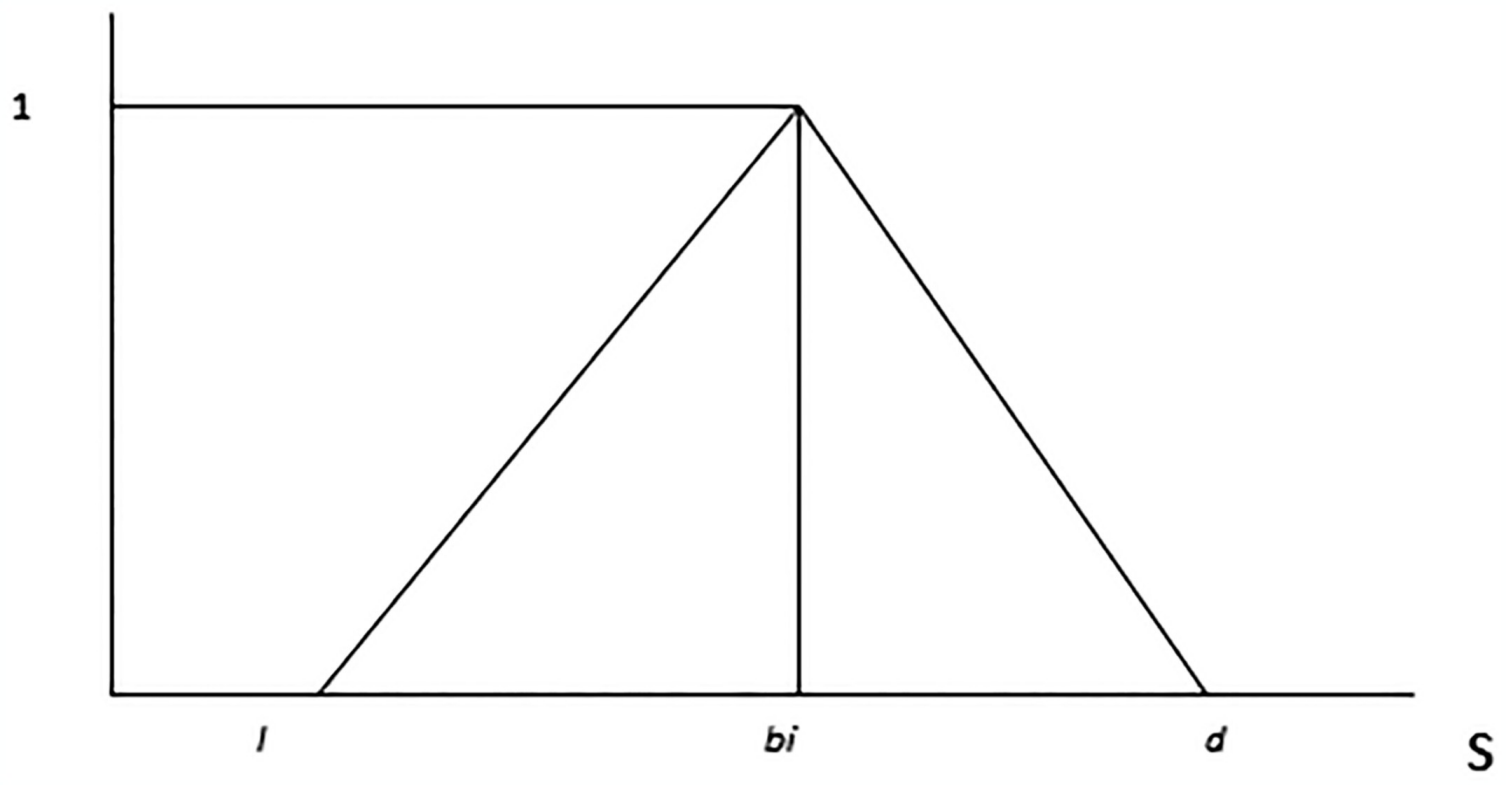
Triangular fuzzy numbers.

In Fig 9, l, bi, and d are defined as the lower, middle, and upper bounds in the triangular membership function, respectively. TFN is denoted by (l, bi, d). For factors that affect values in quantitative terms, experts have assigned scores to the scales presented in Table 2.

**Table 2:** Triangular fuzzy number scale.

Saaty Scale Definition	Fuzzy Triangle Scale
1	Correspondingly significant (1, 1, 1)
3	Inadequately significant (2, 3, 4)
5	Impartially significant (4, 5, 6)
7	Strappingly significant (6, 7, 8)
9	Categorically significant (9, 9, 9)
2	
4	Erratic values between two contiguous measures
6	(1, 2, 3), (3, 4, 5), (5, 6, 7), (7, 8, 9)
8	

Eqs (3)–(6) are taken when converting a numerical value to TFN (Buckley, 1985) and which is expressed as (l_ij_, b_ij_, d_ij_), where l_ij_ is the low value, b_ij_ is the average value, and d_ij_ is the highest value level event. In addition, TFN [ɳ_ij_] is recognized as:

$$\alpha_{\text{ij}}=\left({\text{c}}_{\text{ij}},{\text{bi}}_{\text{ij}},{\text{d}}_{\text{ij}}\right)$$ (3)

where c_ij_ ≤ bi_ij_ ≤ d_ij_

$${\text{c}}_{\text{ij}}=\text{min}\left(\cap_{\text{ijd}}\right)$$ (4)

$${\text{bi}}_{\text{ij}}={\left(\cap_{\text{ij}1},\cap_{\text{ij}2},\cap_{\text{ij}3}\right)}^{\frac{1}{\text{s}}}$$ (5)

$${\text{d}}_{\text{ij}}=\text{max}\left(\cap_{\text{ijd}}\right)$$ (6)

In Eqs (3)–(6), ∩_ijd_ represents the state of the values in comparison between the two factors that practitioner d has given, where i and j stand for two practitioner-determined factors. Based on the geometric mean of professional judgments for particular comparisons, φ_ij_ is calculated. Given the lowest and highest relative relevance scores for the two factors, respectively, the geometric mean may accurately aggregate and express the opinion of the practitioners. Eqs (7)–(9) also support the combined TFN values. Consider two TFNs, M1 and M2, where M1 = (l_1_, mi_1_, u_1_) and M2 = (c_2_, bi_2_, d_2_). Here are the rules for working with them:

$$\left({\text{C}}_{1},\;{\text{bi}}_{1},\;{\text{d}}_{1}\right)+\left({\text{C}}_{2},\;{\text{bi}}_{2},\;{\text{d}}_{2}\right)=\left({\text{c}}_{1}+{\text{c}}_{2},\;{\text{bi}}_{1}+{\text{bi}}_{2},\;{\text{d}}_{2}+{\text{d}}_{2}\right)$$ (7)

$$\left({\text{C}}_{1},\;{\text{bi}}_{1},\;{\text{d}}_{1}\right)\times \left({\text{C}}_{2},\;{\text{bi}}_{2},\;{\text{d}}_{2}\right)=\left({\text{C}}_{1}\times {\text{C}}_{2},\;{\text{bi}}_{1}\times {\text{bi}}_{2},\;{\text{d}}_{1}\times {\text{d}}_{2}\right)$$ (8)

$${\left({\text{C}}_{1},{\text{bi}}_{1},{\text{d}}_{1}\right)}^{-1}=\left(\frac{1}{{\text{d}}_{1}},\frac{1}{{\text{bi}}_{1}},\frac{1}{{\text{c}}_{1}}\right)$$ (9)

Eq (10) is used to create a fuzzy pair-wise comparison matrix in the form of an n x n matrix after obtaining the TFN values for each pair of comparisons. The symbol $\tilde{{\text{B}}^{\text{d}}}$ represents the decision matrix for the pairwise comparisons. This matrix typically contains the fuzzy numbers representing the degree of preference or importance of one element over another in a pairwise comparison.

$$\tilde{{\text{B}}^{\text{d}}}=\left[{\tilde{\text{g}}}_{11}^{\text{d}}{\tilde{\text{g}}}_{12}^{\text{d}}\ldots .{\tilde{\text{g}}}_{1\text{n}}^{\text{d}}{\tilde{\text{g}}}_{21}^{\text{d}}{\tilde{\text{g}}}_{22}^{\text{d}}\ldots .{\tilde{\text{g}}}_{2\text{n}}^{\text{d}}\;\cdots \;\cdots \;\cdots \;{\tilde{\text{g}}}_{\text{n}1}^{\text{d}}{\tilde{\text{g}}}_{\text{n}2}^{\text{d}}\ldots {\tilde{\text{g}}}_{\text{nn}}^{\text{d}}\right]$$ (10)

where (${\tilde{\text{g}}}_{\text{ij}}$) denotes the decision maker’s g^th^ preference for the i^th^ criterion over the j^th^ criterion. If there is more than one decision maker, the average of each decision maker’s preference is obtained using Eq (11).


$${\tilde{\text{g}}}_{\text{ij}}=\sum_{\text{d}=1}^{\text{d}}\;{\tilde{\text{g}}}_{\text{ij}}^{\text{d}}$$ (11)

The next step is to use Eq (12) to update the pairwise comparison matrix $\tilde{\text{B}}$ for all elements of the hierarchy based on the average preference.


$$\tilde{\text{B}}=\lfloor \tilde{{\text{g}}_{11}}\;\ldots \;\tilde{{\text{g}}_{1\text{n}}}\;\cdots \;\ddots \;\cdots \;\tilde{{\text{g}}_{\text{n}1}}\;\cdots \;{\tilde{\text{g}}}_{\text{nn}}\rfloor$$ (12)

Thereafter, the fuzzy geometric mean and fuzzy weight of each factor are described using the geometric mean ${\tilde{\text{Q}}}_{\text{i}}$ method, as shown in Eq (13).


$${\tilde{\text{Q}}}_{\text{i}}={\left(\prod_{\text{j}=1}^{\text{n}}\;{\tilde{\text{g}}}_{\text{ij}}\right)}^{\frac{1}{\text{n}}},\;\text{i}=1,2,3\ldots \text{n}$$ (13)

The next step is to derive the fuzzy weights of the factors using Eq (14). Later, Eqs (15) and (16) are used to compute the mean and normalized weight criteria. Here, ${\tilde{\text{Z}}}_{\text{i}}$ represents the fuzzy weight of the “i”^th^ factor or criterion, and δ_i_ denotes the mean weight of the “i”^th^ criterion. Further, ωr_i_ represents the normalized weight of the “i”^th^ criterion.


$${\tilde{\text{Z}}}_{\text{i}}={\tilde{\text{q}}}_{\text{i}}⊗{\left({\tilde{\text{q}}}_{1}⊕{\tilde{\text{q}}}_{2}⊕{\tilde{\text{q}}}_{3}\ldots .⊕{\tilde{\text{q}}}_{\text{n}}\right)}^{-1}$$ (14)

$$\delta_{\text{i}}=\frac{{\tilde{\text{Z}}}_{1}⊕{\tilde{\text{Z}}}_{2}\ldots ..⊕{\tilde{\text{Z}}}_{\text{n}}}{\text{n}}$$ (15)

$${\omega \text{r}}_{\text{i}}=\frac{\delta_{\text{i}}}{\delta_{1}⊕\delta_{2}⊕\ldots \ldots ⊕\delta_{\text{n}}}$$ (16)

In addition, the center of area (COA) method is used to calculate the BNP value (best fuzzy performance), BNPwD1, of the fuzzy weights in each dimension using Eq (17). Here, dz_1_ represents the upper bound of the fuzzy weight, cz_1_ represents the lower bound of the fuzzy weight, biz_1_ denotes the middle point or center of the triangular fuzzy weight, and Cz_1_ is a constant value representing the center of the area of the triangular fuzzy weight.


$$\text{BNPwD}1=\frac{\left[\left(\text{dz}1-\text{cz}1\;\right)+\left(\text{biz}1-\text{cz}1\;\right)\right]}{3}+\text{Cz}1$$ (17)

**Fuzzy AHP Algorithm**

Enter the initial value from the TFN scale

 construct a pairwise comparison matrix

 evaluate the consistency ratio CR < 0.1

 if not

 repeat

 yes

 calculate an average of preference

 calculate average mean

 weight of the factor

 end

 end

 end

Determining the “average of preference” in the F-AHP method entails examining the relative significance of several elements or criteria in relation to a certain decision. This is accomplished by comparing each element in a pair-wise fashion to every other factor and awarding preference ratings based on their relative value. The “average of preference” is then calculated by taking the mean or average of these preference ratings, which assists in determining the overall preference or priority of each aspect in the decision-making process. The “calculate average mean” stage in F-AHP normally entails aggregating all decision-makers’ or experts’ preference scores or weights assigned to every element. It is a way to obtain a consensus or collective view of the factor’s importance by considering the input from multiple stakeholders. This step helps in achieving a more balanced and representative assessment of each factor’s significance. Ultimately, the procedure culminates in determining the “weight of the factor” in F-AHP. It entails giving each element a numerical weight based on the preference scores derived from the pair-wise comparisons and the determined average mean. The weight represents the contribution of the component to the overall choice and is used to prioritize or rank the factors. Factors with greater weights are thought to be more significant in decision-making, whereas those with lower weights are thought to be less important. This weighting stage ensures that the decision reflects the participants’ pooled preferences and skills, making F-AHP a reliable method for multi-criteria decision analysis.

**Fig 10:**
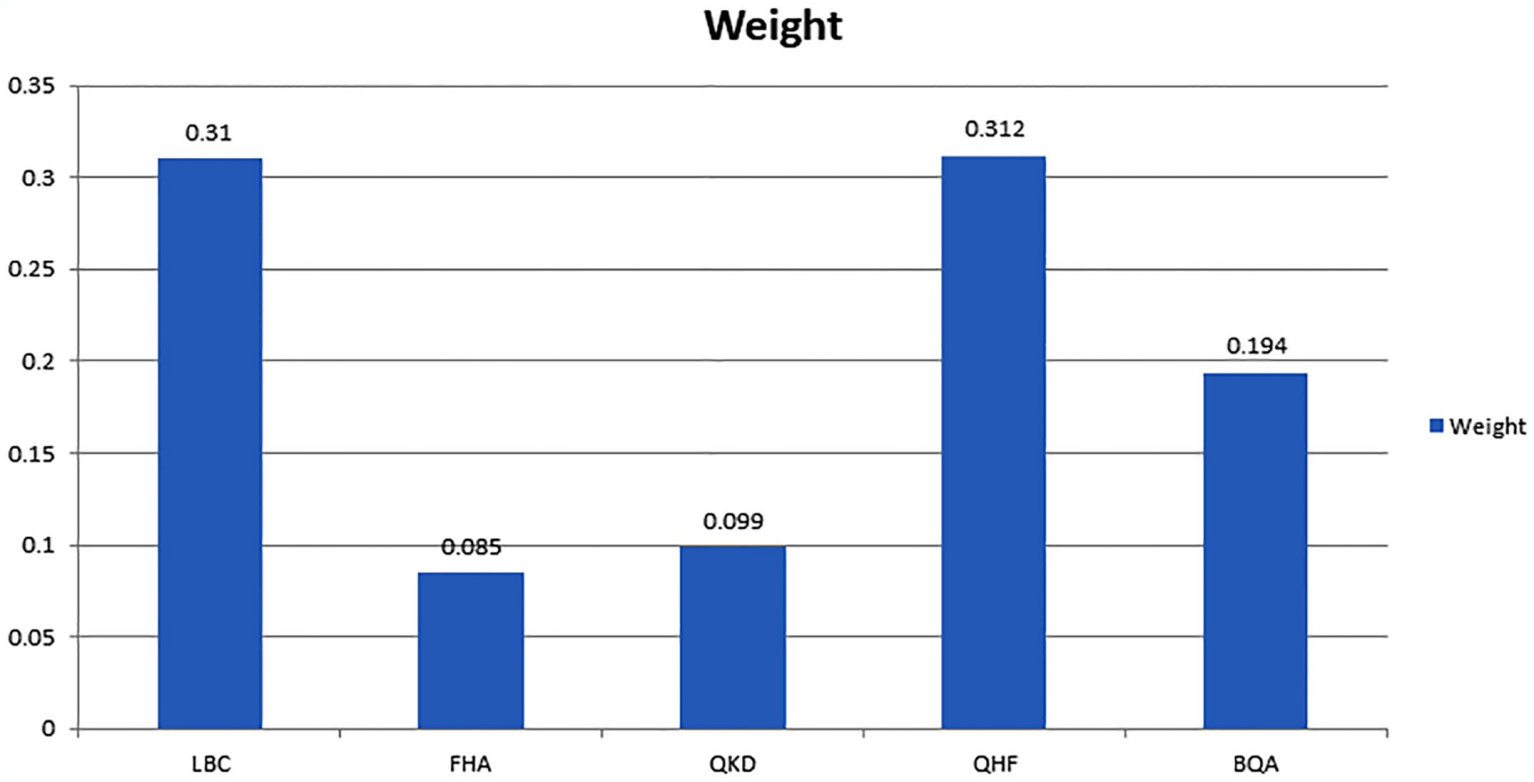
Weight of the quantum techniques.

While the F-AHP methodology is effective for multi-criteria decision making, it does have certain possible issues and limitations. For starters, it places a high value on expert judgement and pair-wise assessments, which might add subjectivity and inconsistency into the decision-making process. It can also be difficult to choose acceptable linguistic phrases and membership functions for ambiguous assessments. Furthermore, as the number of criteria and possibilities increases, so does the analysis’s complexity, necessitating more intensive data collecting and computing. Furthermore, F-AHP may be ineffective in cases with incomplete or unclear information. Sensitivity to alterations in input data and the possibility of differences in outcomes due to varied expert inputs are important factors to consider. Finally, when confronting a large number of criteria or requiring a high level of precision, F-AHP’s application might be resource-intensive. Considering these issues, F-AHP remains a powerful tool for complicated decision-making scenarios when used intelligently and with due attention. Fig 10 illustrates the distribution of weights assigned to various quantum techniques, reflecting their relative importance in the assessment process. Table 3 illustrates the fuzzy pairwise comparison matrix, which captures the relative importance of criteria using fuzzy logic. Tables 4 and 5 present the derived fuzzy and crisp weights, respectively, translating subjective judgments into measurable values. Lastly, Table 6 provides the normalized weights, confirming consistency and comparability in the evaluation process.

**Table 3:** Fuzzy pairwise comparison matrix.

	LBC	FHA	QKD	QHF	BQA
LBC	1.000, 1.000, 1.000	1.000, 2.000, 1.000	3.000, 1.000, 4.000	4.000, 2.000, 1.000	3.000, 5.000, 2.000
FHA	1.000, 0.500, 1.000	1.000, 1.000, 1.000	1.000, 0.111, 0.125	1.000, 2.000, 1.000	0.143, 0.125, 0.333
QKD	0.333, 1.000, 0.250	1.000, 9.000, 8.000	1.000, 1.000, 1.000	1.000, 0.143, 0.167	1.000, 6.000, 3.000
QHF	0.250, 0.500, 1.000	1.000, 0.500, 1.000	1.000, 7.000, 6.000	1.000, 1.000, 1.000	1.000, 6.000, 3.000
BQA	0.333, 0.2000, 0.500	7.000, 8.000, 3.000	1.000, 0.167, 0.333	1.000, 0.167, 0.333	1.000, 1.000, 1.000

**Table 4:** Fuzzy weight.

LBC	[0.3480, 0.3260, 0.2560]
FHA	[0.0760, 0.1030, 0.0760]
QKD	[0.0930, 0.1030, 0.1030]
QHF	[0.2880, 0.2710, 0.3750]
BQA	[0.2010, 0.1970, 0.1830]

**Table 5:** Crisp weight.

LBC	0.3100
FHA	0.0850
QKD	0.0990
QHF	0.3120
BQA	0.1940

**Table 6:** Normalized weight.

LBC	0.3100	2
FHA	0.0850	4
QKD	0.0990	5
QHF	0.3120	1
BQA	0.1940	3
Consistency Ratio (CR)		0.6

## 5. Discussion

The F-AHP multi-measure independent direction approach is utilized to ascertain the weight of each element’s impact. Moreover, with regards to software security from a design standpoint, the efficacy of the software’s security measures is paramount to safeguard against potential breaches or disruptions. This research evaluates different quantum options by combining the input of a thorough literature review with expert opinions to highlight how quantum elements are combined into the Fuzzy AHP framework. The evaluation of different quantum methods is described in detail while following a structured approach in which these technologies are mapped onto the Fuzzy AHP criteria. In this work, by incorporating some of the quantum-specific attributes that include entanglement and superposition into the hierarchical structure of Fuzzy AHP, we ensure these advanced quantum features are weighed properly with conventional security factors. The integration not only comes out to enhance the assessment concerning relevance about quantum-resilient security but defines a much more accurate and articulate insight into how quantum techniques influence the overall software security outcomes. This investigation chose five significant quantum security techniques. The results indicate that quantum security algorithms or procedures exert varying degrees of influence on the software in diverse applications. According to our evaluation, which used the F-AHP approach, the component of the example quantum hash function for the quantum technique received the principal rank. Lattice-based cryptography received the second weight of the quantum technique. We evaluated the components and their dependent choices to analyze the impact of the quantum technique. As a result, we approached the system that could combine F-AHP to arrange the results according to the order. The following are the key findings about quantum techniques in applications for software security:

- The results of the quantum technique evaluation for software security will provide a credible reference for engineers and scientists working in a similar domain. This study’s empirical research drew a methodically aligned and precise list of the most significant design factors influencing quantum security.
- The factor that received the maximum attention was the quantum hash function in the evaluated outcome of the quantum technique for securing software.
- Second place was taken by the quantum technique’s lattice-based cryptography.

The quantum technique is impacted by several issues (due to threats). For developers and maintenance specialists, any error in a piece of software poses a huge problem that could result in data loss and financial losses. Therefore, it is crucial from a design perspective to concentrate on methods that would lower the security risk to software in the era of quantum computing. In this research study, the variables and their related alternatives were explored to attain the proper level of security in software. Our investigation demonstrated that the quantum hash function should be given the top priority in software security. In this study, the weights of the components and alternatives found through the empirical analysis were measured by using the fuzzy-based technique of AHP.

The F-AHP multi-measure autonomous direction procedure was used to validate the findings of this research investigation. It created a credible reference for scientific researchers and engineers in the area of software security by evaluating the impact weights of different quantum security solutions. This study carefully ranked the most relevant quantum security design features, with the quantum hash function receiving the most attention, with lattice-based cryptography coming in second. These findings provide important insights for minimizing software security vulnerabilities, especially in the age of quantum computing. The validation of the findings using the F-AHP approach confirmed their dependability, making them a valuable resource for software security specialists and opening up new options for future research as the field progresses.

## 6. Conclusions

The introduction of quantum computing has resulted in a paradigm change that necessitates a thorough re-evaluation of software security mechanisms in the rapidly growing field of information technology. This study has shown that the quantum approach, which includes important methods like the lattice-based cryptographic algorithm (LBCA), the fully homomorphic algorithm (FHA), quantum key distribution (QKD), the quantum hash function (QHF), and the blind quantum algorithm (BQA), is a key pillar in addressing the growing complexity of software security. We have thoroughly assessed the effects of these quantum security approaches by utilizing the fuzzy analytic hierarchy process (F-AHP) methodology. The quantum hash function was categorically identified as a major influencer by our empirical investigation, which was supported by its crucial function in strengthening software security. Lattice-based encryption also established itself as a strong companion in the quantum security domain, emphasizing its significance. With the development of quantum computing, the need for software security is becoming more and more urgent. Software developers, specialists, and security experts must not only understand the complex complexities of quantum security approaches but also make wise selections according to their relative impact because the ramifications of a breach are extensive. Our research offers a proven and systematic method for evaluating the importance of various quantum security components, serving as a solid platform for such decision-making. The insight gained from the present research can not only direct the present software security initiatives but also pave the way for further research into how to address the constantly changing problems brought on by the quantum computing revolution. Our results demonstrate the crucial importance of quantum approaches in strengthening the foundations of software security in the quantum age, where quantum physics and security requirements interact dynamically. Future research needs to focus on the development and refinement of quantum-resistant algorithms in view of evolving quantum threats. How these algorithms can be fitted into existing systems, and then under real-life conditions, has to be investigated. Besides, one needs to account for the transition challenges from classical to quantum-resistant security measures, such as computation overhead and interoperability issues, to find viable solutions. Any future research will also have to take into consideration how quantum computing affects new technologies and standards that are emerging, in such a manner that quantum-resilient strategies can be adaptable in an infinitely changing technological environment. These bring a path for researchers toward robust and forward-looking strategies of quantum-enhanced security in software.

## Abbreviations

AHP: Analytic hierarchy process
BQ: Blind Quantum
BQA: Blind quantum algorithm
BQA: Blind Quantum Algorithm
COA: Center of Area
CR: Consistency Ratio
F-AHP: Fuzzy analytic hierarchy process
FHA: Fully Homomorphic Algorithm
LBCA: Lattice-Based Cryptographic Algorithm
LLL: Lenstra–Lenstra–Lovász lattice-reduction algorithm
MCDM: Multi-Criteria Decision Making
QHF: Quantum hash function
QKD: Quantum key distribution
QuPUFs: Quantum Physically Unclonable Functions
RSA: Rivest–Shamir–Adleman (cryptography)
SSDLC: Secure software development lifecycle
SVP: Shortest vector problem
SVP: Shortest Vector Problem
TFN: Triangular Fuzzy Number
